# Guardians' Subscriptions to Voluntary Hospitals

**Published:** 1905-01-28

**Authors:** 


### GUARDIANS' SUBSCRIPTIONS TO VOLUNTARY HOSPITALS.

It is common in many places for the guardians of the poor to give an annual subscription to the voluntary hospital or hospitals in the neighbourhood. Thus the parish of Paddington gives 10 guineas to St. Mary's Hospital, and the Wandsworth Guardians give £112 annually in subscriptions to different hospitals in London and elsewhere. The reason for these donations is that the voluntary hospitals receive many patients who otherwise would have to be taken into the Poor-law infirmaries, and the guardians thus acknowledge their appreciation of the benefit received. The practical reasonableness of this is obvious, yet it must be admitted that it raises a delicate point in the relations between paupers and non-paupers, voluntary and rate-supported hospitals. Many of our Poor-law infirmaries are to all intents and purposes ordinary general hospitals. They are well-equipped, they have trained nurses, and their patients are drawn from the same class of society as those who go to the hospitals. The infirmary takes chronic cases which a hospital would refuse, but often there is little else to differentiate the one from the other. In cases of accident, it is usually a matter of the comparative proximity of one or the other which decides whether a patient shall be taken to the hospital or to the Poor-law infirmary. Thus patients of a respectable and even a superior class find their way to the infirmary, people whom it would be absurd to label "pauper," yet paupers they are, strictly speaking, while they are in the institution, and subject after they leave it to all the disabilities of those who have accepted Poor-law relief. A casuistic mind might argue that the same disability should apply to the patients in any hospital which receives rate-aid in the form of a subscription from the local guardians, even though this form but a microscopic fraction of the cost of maintenance. The disabilities of the infirmary patient come out in strange ways. Thus convalescent institutions refuse to receive paupers. Thus it may happen that of two men—friends, neighbours, fellow-workmen—who have been ill, one, who had the luck to get into a voluntary hospital, may complete his cure at some healthful country place, while the one who was treated in the infirmary has to return straight home, unless the guardians have made arrangement for the boarding of their convalescents at some of the places—few in number and not always well managed —which take in paupers. As our Poor-law infirmaries continue to improve, these inconsistencies are likely to increase. As it is, it happens that in some cases Poor-law infirmaries take paying patients, or at least reserve the right to charge a patient any sum up to the full cost of his maintenance. It is a little hard that a man who is paying for all he receives should be dubbed a pauper while another may get his treatment free, yet escape the stigma. These facts may be taken as good ground for the contention that the voluntary hospitals and Poor-law should actively co-operate together in the interests of everybody. There is another aspect which should have weight with the ratepayers. Voluntary hospitals are provided for the poor out of the pockets of the rich. To the Poor-law infirmaries all ratepayers, rich and poor alike, subscribe, *nolens volens*, and are liable to punishment if they fail to do so. It is, therefore, to the interest of the poorer ratepayers that voluntary hospitals should continue to exist, and we regard the donations of guardians as an admission of this fact.

